# Development of a Smartwatch with Gas and Environmental Sensors for Air Quality Monitoring

**DOI:** 10.3390/s24123808

**Published:** 2024-06-12

**Authors:** Víctor González, Javier Godoy, Patricia Arroyo, Félix Meléndez, Fernando Díaz, Ángel López, José Ignacio Suárez, Jesús Lozano

**Affiliations:** Industrial Engineering School, University of Extremadura, 06006 Badajoz, Spain; victorgb@unex.es (V.G.); jgodoydz@unex.es (J.G.); parroyoz@unex.es (P.A.); felixmv@unex.es (F.M.); fdiazgrc@unex.es (F.D.); angellopez@unex.es (Á.L.); jmarcelo@unex.es (J.I.S.)

**Keywords:** metal oxide semiconductor sensor, smartwatch, carbon dioxide, methane, toluene, xylene, ethylbenzene, principal component analysis, prediction

## Abstract

In recent years, there has been a growing interest in developing portable and personal devices for measuring air quality and surrounding pollutants, partly due to the need for ventilation in the aftermath of COVID-19 situation. Moreover, the monitoring of hazardous chemical agents is a focus for ensuring compliance with safety standards and is an indispensable component in safeguarding human welfare. Air quality measurement is conducted by public institutions with high precision but costly equipment, which requires constant calibration and maintenance by highly qualified personnel for its proper operation. Such devices, used as reference stations, have a low spatial resolution since, due to their high cost, they are usually located in a few fixed places in the city or region to be studied. However, they also have a low temporal resolution, providing few samples per hour. To overcome these drawbacks and to provide people with personalized and up-to-date air quality information, a personal device (smartwatch) based on MEMS gas sensors has been developed. The methodology followed to validate the performance of the prototype was as follows: firstly, the detection capability was tested by measuring carbon dioxide and methane at different concentrations, resulting in low detection limits; secondly, several experiments were performed to test the discrimination capability against gases such as toluene, xylene, and ethylbenzene. principal component analysis of the data showed good separation and discrimination between the gases measured.

## 1. Introduction

Measuring air quality in both industrial and domestic environments is crucial for safeguarding human health and for environmental sustainability. Industrial activities release pollutants that can pose health risks, while indoor environments, generally poorly ventilated, may harbor harmful compounds. Precise monitoring and control of air quality can help identify the hazards to which people are exposed on a daily basis and, consequently, would allow the implementation of strategies that could correct the adverse effects of poor air quality, thus ensuring healthy living and working conditions. 

The production of toxic and hazardous gases is very common in industrial environments. Some chemical agents, such as toluene, xylene, or ethylbenzene, are in the spotlight of researchers because they are widely used as solvents in different industrial processes [1]. Exposure to these may adversely affect human health. For example, prolonged exposure to toluene has been associated with motor incoordination, dizziness, relaxation, and light-headedness [2]. High exposure to xylene can damage liver function, as it is mainly metabolized in the liver. Moreover, the intensity of the health effects produced by xylene is determined by the exposure medium and the duration [3]. Furthermore, ethylbenzene exposures are also related to liver damage, and ethylbenzene-associated reactive oxidative metabolites are linked to carcinogenesis [4]. 

It is also common in industry to use methane (CH_4_) as an energy source [5]. In its combustion reaction, one molecule of CH_4_ produces one molecule of carbon dioxide (CO_2_), as detailed below.

(1) $${CH}_{4}+2O_{2}\to {CO}_{2}+2H_{2}O$$

Carbon dioxide is associated with global warming and environmental degradation [6]. Exposures to CO_2_ above 500 ppm have been related to an increase in blood pressure and an increase in heart rate; higher concentrations above 1000 ppm and 10,000 ppm are related to cognitive problems and an increase in respiratory rate, respectively [7]. On the other hand, although CH_4_ is usually harmless, high concentrations can cause suffocation since it displaces oxygen in a closed space; in addition, it can also produce explosions when concentrations reach 5% to 15% in the air since it is a flammable gas [8].

In Spain, the document on Occupational Exposure Limits for Chemical Agents includes the safety values of chemical agents adopted by the National Institute for Safety and Health at Work and approved by the National Commission for Safety and Health at Work [9]. Specifically, this document establishes two important Environmental Limit Values (ELVs): daily exposure (ELV-DE) and short-term exposure (ELV-SE). Firstly, the ELV-DE value represents the average concentration of the chemical agent in the breathing zone of the worker measured or calculated on a time-weighted basis for the actual working day and refers to a standard eight-hour working day. Secondly, the ELV-SE value is the average concentration of the chemical agent in the breathing zone of the worker, measured or calculated for any 15-min period throughout the workday. Following these definitions, Table 1 shows the respective ELV for toluene, ethylbenzene, xylene, CO_2_, and CH_4_. 

To measure air quality, reference stations are usually installed at fixed locations within a city or region. The data received from these stations are used to construct spatio-temporal air quality maps. However, their resolution is low mainly due to two problems [10]: one, the low number of stations (due to their high cost and maintenance), and the other, the low sampling frequency of the sensors. That is the reason why portable personal air quality detection systems are gaining importance in recent times. Although these devices are less accurate than reference stations, they have the ability to increase the spatio-temporal resolution and, because of their low cost, they can be deployed ubiquitously. Their applications are not only limited to industrial environments, but they are also used to learn more about how people are exposed to air pollution in the indoor and outdoor spaces where they carry out their daily activities, so that they can act in an informed way to reduce their exposure to air pollution [11].

Gas sensor fabrication is carried out using different technologies [12], including optical, surface acoustic wave (SAW), electrochemical, catalytic and semiconductor-based technologies such as those used in metal oxide (MOX) sensors. MOX sensors are increasingly used due to their low cost, short response time, and their wide range of target gases. Basically, they consist of a heated surface of a metal oxide, which changes its electrical resistance depending on the oxygen content of the surface; depending on the semiconductor materials, they can be classified as p-type or n-type semiconductor compounds. The presence of oxidizing gases, such as NOx, increases the resistance, while reducing gases, such as volatile organic compounds (VOCs), reduce the resistance. Humidity also affects the sensor, as water vapor can lead to chemical adsorption; this can generate OH groups and electrons which are released into the conduction band of MOX sensors. Thus, this adsorption can cause a reduction in sensor resistance [13].

The use of MOX sensors in portable equipment for measuring air quality is increasingly popular. They are proving to be reliable in detecting the main pollutant gases (such as CO, NO_2_, and O_3_) in cities [14], BTEX compounds (benzene, toluene, ethylbenzene, and xylenes) released into the environment through transportation and many industrial manufacturing processes and other industrial gases [15,16,17]. Also, they have been used for developing pollutant maps and monitoring indoor air quality [18,19]. The use of robots is also a point of interest because it allows the automation of sampling for air quality measurement without the need for human supervision [20]. 

This paper presents a novel device that integrates different MOX sensors inside a smartwatch. The main objective is to test the sensing capabilities of the system in laboratory conditions using toluene, ethylbenzene, xylene, CO_2_, and CH_4_. Firstly, limit detection analyses with different concentrations of CO_2_ and CH_4_ are presented to test the detection capabilities of the device using the values shown in Table 1 as a reference. Finally, the discrimination capabilities of the smartwatch were tested using toluene, xylene, and ethylbenzene using principal component analysis (PCA) for dimensionality reduction. 

The main advantage of the proposed device is its wearable capability, as the sensors are implemented inside a smartwatch. In addition, the device is easy to use, making it optimal for any user, whether experienced or non-experienced, to monitor the air quality of their surroundings. 

## 2. Materials and Methods

### 2.1. Gas Samples

Compressed CH_4_ and CO_2_ 5000 ppm balanced in N_2_, and synthetic air (N_2_: 50–80% and O_2_: 20–50%) gas bottles provided by NIPPON GASES ESPAÑA S.L.U (Madrid, Spain) were utilized. On the other hand, permeation tubes for toluene, xylene, and ethylbenzene were home fabricated.

**Figure 1:**
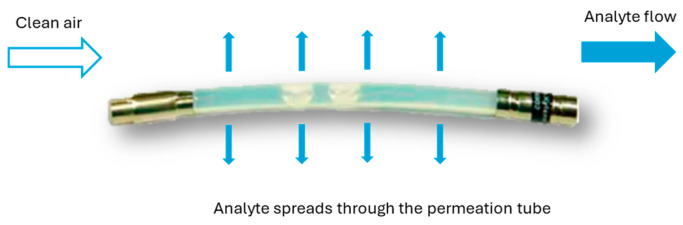
Permeation tube diffusion process.

Permeation tubes are polymeric tubes, normally PTFE (Polytetrafluoroethylene), that contain a solid or liquid chemical compound and are sealed and crimped in both extremes. As can be seen in Figure 1, the chemical compound permeates through the tube walls at a constant permeation rate at a given temperature. Then, the chemical compound mixes and is transported by a diluent gas [21].

Permeation tubes were built in the laboratory and calibrated using a vapor generator OVG-4 from Owlstone (Cambridge, UK) with an integrated heater programmed at a reference temperature of 100 °C. Permeation tubes were built following the instructions given by [22]. One-quarter inch PTFE tubes were used. The tubes were crimped on one side, and after that, 1 mL of the chemical compounds were placed inside the tube. Finally, the tube was crimped in the other extreme.

Each permeation tube was weighted periodically to obtain the permeation rate of the tube, and the permeation rate was obtained as the slope of the curve Mass loss vs. Time. Permeation rates obtained for toluene, xylene, and ethylbenzene are, respectively, 25,891.23 ng/min, 11,927.35 ng/min, and 6959.67 ng/min.

### 2.2. Description of the Smartwatch

**Figure 2:**
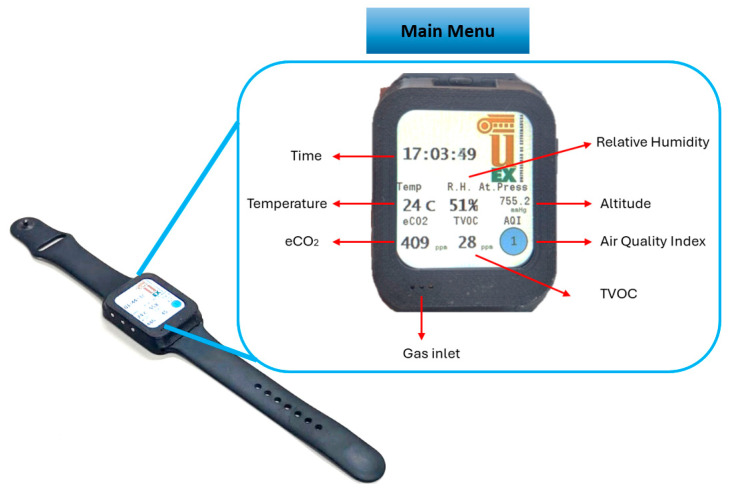
Smartwatch design and its main menu.

The smartwatch developed is shown in Figure 2. Externally, the design consists of a Polylactic acid (PLA) housing that accommodates the electronics, the battery and a liquid crystal display (LCD) screen to display sensor and air quality information. In addition, the case is provided with gas inlet holes to ensure a good absorption/desorption. A silicone elastic band was attached to the case to fasten the watch to the wrist. Furthermore, three push buttons were included to navigate through different sensor menus and to set the time.

The main menu shown in Figure 2, apart from the time, displays different sensor values, temperature, relative humidity, altitude (expressed in pressure units), equivalent CO_2_ (eCO_2_), total volatile compounds (TVOC), and an ambient air quality index (AQI). The eCO_2_ and AQI values are obtained from the TVOC information. The air quality index is represented visually in such a way that it can be easily interpreted by inexperienced users. AQI values are calculated in accordance with the German Federal Environmental Agency guidelines [23]. They are represented by circles that change in color according to the surrounding air quality. Table 2 shows the detailed interpretation of the meaning of each color; the interpretation of these values is obtained from ENS160 datasheet [24]. 

**Figure 3:**
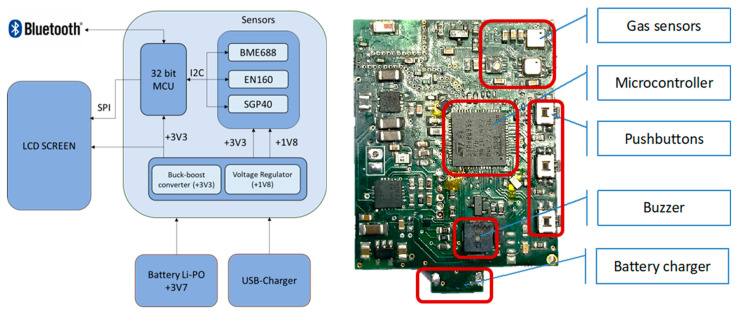
Smartwatch block diagram (**left**) and electronic board (**right**).

Figure 3 depicts, on the one hand, the internal block diagram of the electronics of the smartwatch and, on the other hand, an actual image of its electronic prototype. As can be appreciated in the diagram on the left, the watch integrates three MOX-type sensor modules: the BME688 from Bosch (Gerlingen, Germany), the SGP40 from Sensirion (Stäfa, Switzerland), and the ENS160 from ScioSense (Eindhoven, The Netherlands). These sensors are connected via an I^2^C bus to an ultra-low-power 32-bit microcontroller (STM32WB55 model) from STMicroelectronics (Geneva, Switzerland). This chip integrates two cores, a 64 MHz Arm Cortex-M4 and a 32 MHz Arm Cortex-M0+, 1 Mbyte of Flash memory, a Bluetooth Low Energy 5.4 module for wireless communications and an SPI interface where the LCD display is connected. The microcontroller is responsible for data acquisition from the sensors via the I^2^C bus and forwarding this information via Bluetooth to a smart device for archiving and further processing and analysis. The entire system is powered by a 3.7 Vdc lithium polymer battery that allows the smartwatch to be used for 9 h continuously and autonomously. This battery is rechargeable via a USB connection. In addition, a +3.3 Vdc step-down converter and a +1.8 Vdc linear regulator are used to power the microcontroller and sensors. 

Table 3 summarizes some information about the sensor modules and the signals they provide [24,25,26]. The BME688 is a digital gas sensor that allows measurements of temperature, pressure, humidity and gas resistance. ENS160 is a semiconductor module with different MOX-type sensor elements. Each element has an independent hotplate to detect different compounds. It incorporates internal algorithms to process raw sensor signals to obtain TVOCs, eCO_2_, and AQI values. In addition, according to the manufacturer, the algorithms themselves can compensate for the effects of relative humidity and temperature on these values. Finally, the SGP40 is a MOX-based gas sensor that also measures raw values proportional to the logarithm of the resistance of the sensing material. Also, it uses an integrated hotplate to compensate for the effects of the relative humidity.

### 2.3. Measurement Setup

Given the use of different types of vessels in the measurements, two distinct experimental setups are employed. The first setup utilizes gas bottles, while the second setup is designed for permeation tube measurements; all the setups were configured for laboratory measurements.

#### 2.3.1. Gas Bottle Measurement Setup

**Figure 4:**
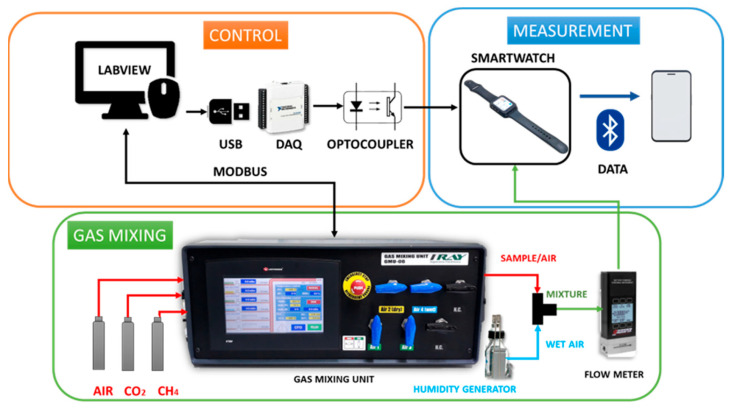
Experimental setup for gas bottles.

To measure gases stored in bottles, the setup used can be observed in Figure 4. Three different parts can be found in this setup: the control stage, the gas mixing stage, and the measurement stage.

Firstly, the control stage is responsible for communicating with the gas mixing unit and the smartwatch. A National Instruments LabVIEW (Austin, EEUU) application is used to control the gas mixing unit, communication between them is made by MODBUS. LabVIEW application allows the configuration of the total flow of the system, the concentration of the sample gases, the relative humidity and sets the absorption (sample) and desorption (air) time for measurements. In addition, a data acquisition card (DAQ) is used to send digital pulses to the smartwatch when the program changes between adsorption and desorption time.

Secondly, the gas mixing stage is responsible for creating the mixtures necessary to make the measurements. It takes dry air and mixes it with the samples. Then, a mixture is created when sample/air flows through a humidity generator. Output gas is the sum of the percentage of humid gas configured, the concentration of the sample, and the rest is dry air. A flow meter is used to check that the total flow configured is correct.

Finally, in the measurement stage, the printed circuit board of the smartwatch, which is located inside a methacrylate cell with dimensions of 50 × 139 × 13 mm^3^, is exposed to generated outgas; the cell was also located in an extractor hood to eliminate the gases from the setup. The smartwatch is responsible for sending data from the gas sensors to a smartphone for future data processing.

#### 2.3.2. Permeation Tube Measurement Setup

**Figure 5:**
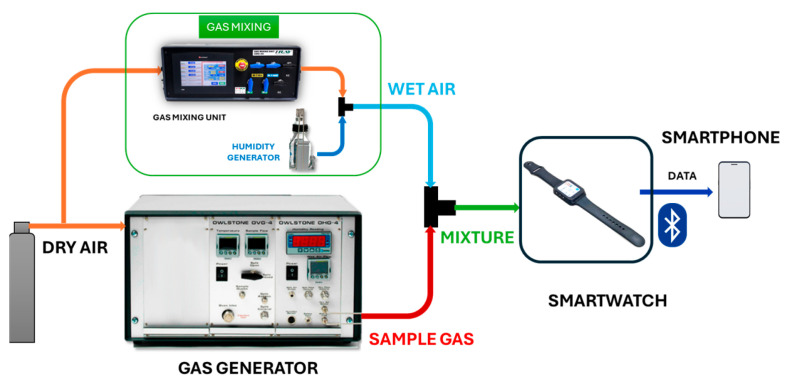
Permeation tube measurement setup.

The setup made for measuring permeation tubes is observed in Figure 5. In this setup, a gas generator OVG-4 from Owlstone is used. The same control stage explained in the last section is used, as well as the gas mixing unit to generate humid gas.

Permeation tubes are introduced into the gas generator, and the desired concentration is adjusted by varying the temperature of a heater integrated into it. As can be observed in Figure 5, dry air is introduced into the gas generator and it passes through the permeation tube generating the desired concentration. Furthermore, dry air is also introduced into the gas mixing unit to generate a certain amount of humid air. Finally, the sample gas at the desired concentration and the humid air generated are mixed in a T-joint in the absorption time, while in desorption time, only humid air is introduced into the smartwatch.

### 2.4. Limit of Detection Measurements

All the smartwatch sensor modules were exposed to 9 different concentrations of CO_2_ and CH_4_ to test the detection capabilities. CO_2_ concentrations generated were from 3000 ppm to 50 ppm, so the measurement range meets the values shown in Table 1 and even lower to test the detection capabilities at low concentrations. CH_4_ concentrations were generated from 1000 ppm to 10 ppm. Measurements were completed with an absorption time of 2 min and a desorption time of 4 min. Samples were taken every 2 s, so the total measurement time was 3478 s, humid air was generated at 40% as regular indoor value, and the total flow programmed was 100 mL/min.

### 2.5. Discrimination Capabilities Measurements

The sensor modules were tested with three different gases: toluene, xylene, and ethylbenzene. The concentrations generated and the programmed temperature to reach them can be seen in Table 4. These values are obtained according to Equation (2) [21].

(2) $$C=\frac{24.45\cdot q_{d}}{M\cdot Q}$$

where *C* is the target concentration in ppm, *M* is the molar weight of the gas in g/mol and *Q* is the total flow in mL/min (sample flow and humid flow) whose value was adjusted at 150 mL/min, and *q_d_* is the permeation rate of the tubes at working temperature in ng/min. As *q_d_* is dependent on temperature when the working temperature is different from the calibration temperature, it is necessary to correct it with Equation (3).

(3) $$q_{d}=q_{d,calibration}e^{-6794\left(\frac{1}{T}-\frac{1}{T_{calibration}}\right)}$$

Concentration values were generated below the ones shown in Table 1 to test the discrimination capabilities at low concentrations. Sampling time and relative humidity were the same as in Section 2.4. In addition, 10 repetitions were made at each absorption/desorption cycle.

Measurement cycles were configured as follows. The desorption cycle was configured with 60 mL/min of humid air and 90 mL/min of dry air. A desorption time of 8 min was selected. The absorption cycle was configured with 60 mL/min of humid air, 90 mL/min of sample gas and 4 min to ensure a stationary response of the sensors. The total time of measurements was 7676 s.

### 2.6. Data Analysis

A total of 10 different variables were measured among all the modules. Detailed information on each variable can be found in Table 5.

For each repetition made when discrimination capabilities are tested, it is necessary to take a characteristic value of each sensor response. This characteristic value (CV) was obtained according to Equation (4).

(4) $$CV=\left(V_{max}-V_{min}\right)\times 100-1$$

where *V_max_* is the peak of each repetition made and *V_min_* is the minimum value obtained in the measurement. Processed data are stored in a matrix of characteristic values, where each column represents a variable from the sensors and each row stores the values obtained for a given measure. Thus, the matrix dimensions are 30 × 10, corresponding to the 10 variables from the sensors and the 10 repetitions of the 3 measured gases. However, the first repetition of each gas is discarded as the sensors’ response is not yet stabilized. Furthermore, data from temperature and relative humidity are not used as they are not variated in the experiment.

As a large amount of data is obtained when the discrimination capabilities of the smartwatch are tested, mathematical algorithms are needed to process all the data information. Dimensionality reduction of all the datasets from the matrix of characteristics values was applied using principal component analysis (PCA). With this technique, the dataset is described with new non-correlated variables, also known as components, and make it possible to visualize them in a plot. These components are classified by the amount of the original variance they represent [27].

To see what variables of the sensors are more relevant to each component, load plots were used. The algorithm used for this analysis is explained in [28]. This algorithm returns the coefficients of the principal components also know as loads. Each row of the matrix of coefficients contains the coefficients of a principal component while the number of columns corresponds to the number of variables.

High values (negative or positive) from loads indicate that the variables have a strong influence on that component. However, if a load is close to zero, that variable has a negligible influence on that component.

## 3. Results and Discussion

### 3.1. Limit of Detection Measurements

**Figure 6:**
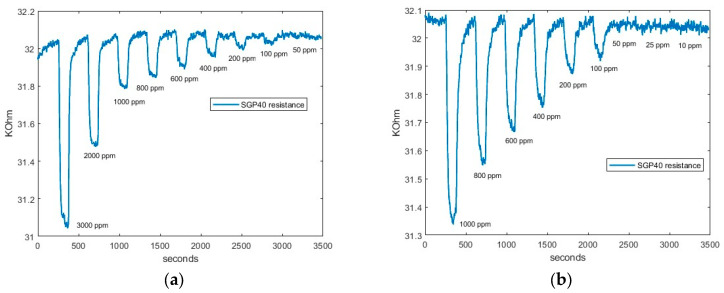
SGP40 response: (**a**) SGP40 CO_2_ response; and (**b**) SGP40 CH_4_ response.

**Figure 7:**
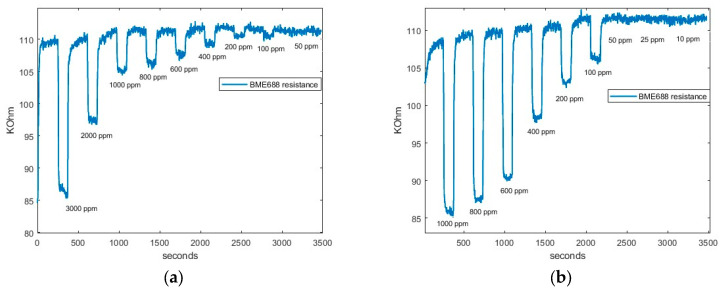
BME688 response: (**a**) BME688 CO_2_ response; and (**b**) BME688 CH_4_ response.

**Figure 8:**
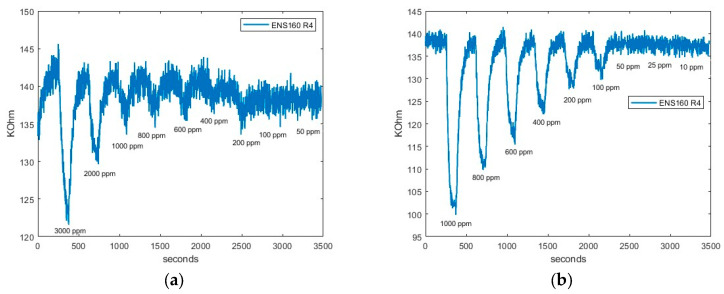
ENS160 response: (**a**) ENS160 CO_2_ R_4_ response; and (**b**) ENS160 CH_4_ R_4_ response.

The limit of detection results of SGP40, BME688, and ENS160’s fourth resistance (R_4_) when CO_2_ and CH_4_ are measured are presented in Figure 6, Figure 7, and Figure 8, respectively.

Firstly, as can be seen in Figure 6a, SGP40 can detect all the concentrations up to 100 ppm of CO_2_. In addition, Figure 6b shows the response when CH_4_ is measured. The sensor is also capable of detecting concentrations above 50 ppm.

Secondly, in Figure 7, the BME688 response exhibits a detecting range similar to SGP40, reaching a limit detection for CO_2_ and CH_4_ above 50 ppm. Finally, in Figure 8, the ENS160’s fourth resistance (R_4_) response shows a lower limit detection than BME688 and ENS160, being capable of detecting concentrations different from baseline approximately up to 400 when CO_2_ is measured. However, when CH_4_ is measured, the minimum value detected is 100 ppm.

From all responses, a clear tendency can be seen. When synthetic air is exposed to the smartwatch (desorption), the resistive value increases; this value corresponds to the baseline of the sensors. However, when sample gas is applied, resistive values decrease. In addition, it can be observed that the higher the concentration is, the higher the difference between the maximum and the minimum value reached in the cycle. Thus, a more significant resistive value is reached with higher concentrations.

To obtain the limit of detection (*LOD*), the data from resistance were used because other data provided by the sensors are calculated with algorithms that could not provide a linear response. The formula used to obtain the limit of detection of the sensors is presented in Equation (5) [29,30], where s_0_ is the standard deviation of the of the blank measurements (desorption measurements) and $\hat{A}$ is the slope of the resistive response of the sensors. To obtain the regression equations of the resistive response, Equation (4) was used as characteristic value on each measurement.

(5) $$LOD=\frac{3.3\cdot s_{0}}{\hat{A}}$$

**Figure 9:**
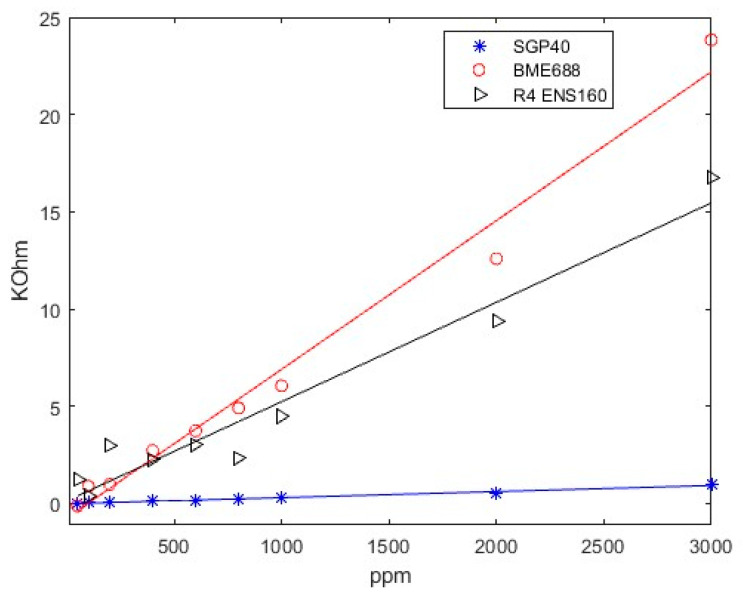
Lineal regression on CO_2_ response.

**Figure 10:**
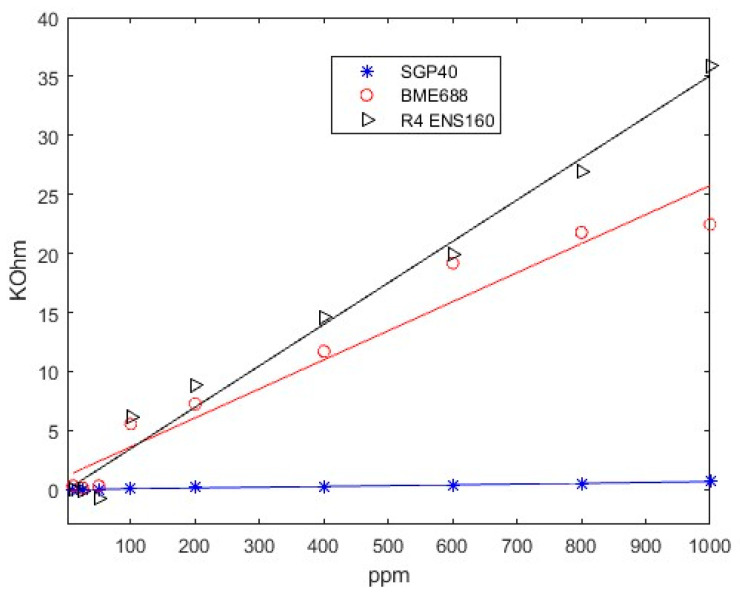
Lineal regression on CH_4_ response.

Figure 9 and Figure 10 shows as an example the regression made on SGP40, BME688, and R_4_ ENS160; the response is approximately linear over the whole range of concentration. That is, higher concentrations give a better linear response. However, lower concentrations give a worse response as the variations of resistance are less differentiated from the baseline. The fitted equations of all sensors, R^2^ parameters and *LOD* for each gas can be seen in Table 6 and Table 7. The results show a good linear approximation, with R^2^ parameters greater than 0.9, so the fitted equations could give a good prediction on the resistance values given a concentration value. In addition, the *LOD* obtained with Equation (5) is consistent with the values observed in Figure 6, Figure 7 and Figure 8.

### 3.2. Discrimination Capabilities Measurements

**Figure 11:**
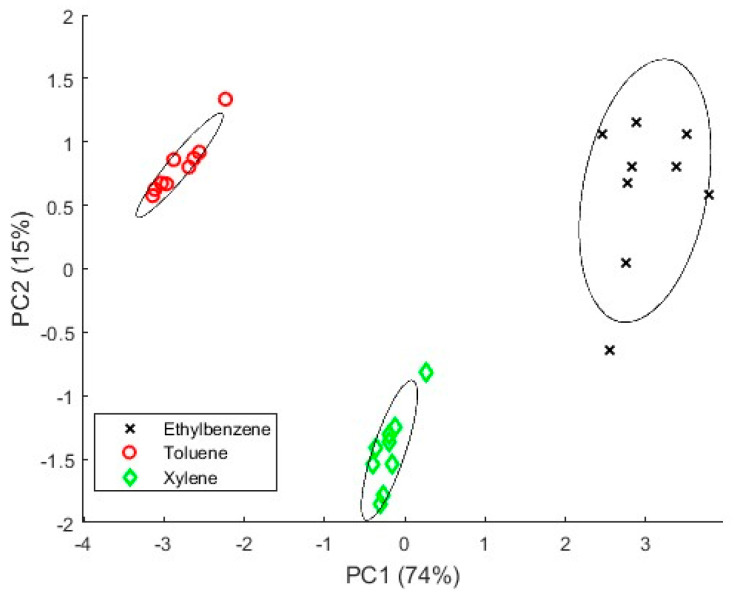
PCA analyses when ethylbenzene, toluene, and xylene are measured.

A PCA analysis was performed to understand the discrimination capabilities when toluene, ethylbenzene, and xylene are measured. Figure 11 shows the PCA plot, where the first component (PC1) has an explained variance of 74% while the second component (PC2) has 15% of explained variance. As can be seen, three different groups are well separated, corresponding to the three gases measured.

**Figure 12:**
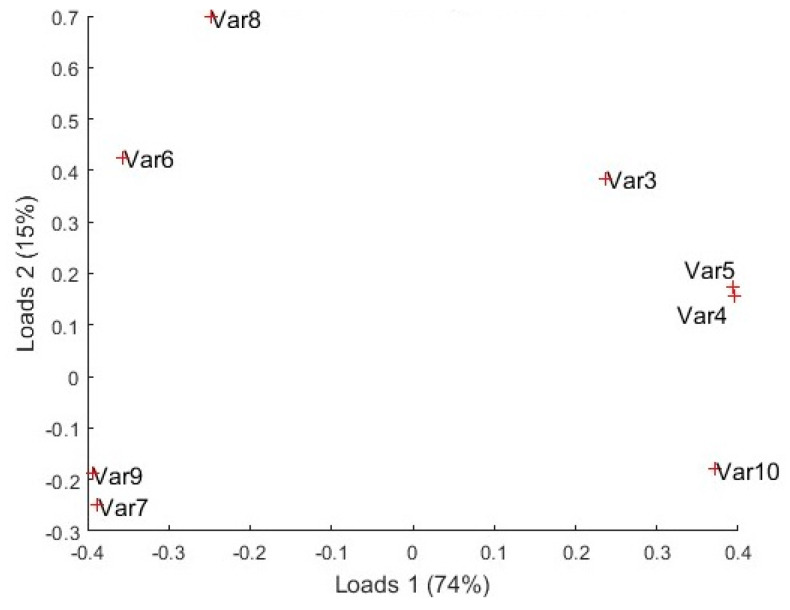
Load plots.

If the load plot is studied (Figure 12), all variables contribute to the separation in component 1. However, the ones with more relevance in the positive part of component 1 are Var3, Var4, and Var5, corresponding to the ENS160 resistive values, and Var10, corresponding to the BME688 resistance. In addition, in the negative part of component 1, the more relevant variables are Var9, Var7, and Var6, corresponding to eCO_2_, TVOCs, and AQI of ENS160, respectively; Var8 also has relevance corresponding to the SGP40 resistance value.

## 4. Conclusions

In this work, a novel smartwatch that integrates gas sensors is presented. The device is intended to be user-friendly and portable. The smartwatch includes different non-specific commercial MOX sensor modules and allows the detection of different gases.

In addition, detection and discrimination capabilities with different industrial gases were tested in laboratory conditions. Firstly, different concentrations of CO_2_ and CH_4_ were measured to test the detection capabilities, and the device was able to detect lower concentrations than the minimum shown in Table 1. However, concentrations of these gases below 100 ppm could not be differentiated from baseline. Also, the device was proven to have a good linear response in the whole range of measurements, providing a good prediction model.

Secondly, the discrimination capabilities of the device were tested with toluene, xylene, and ethylbenzene. PCA results showed that the smartwatch can discriminate the three different gases at a very low concentration. Therefore, it could be helpful in warning workers of leakages with concentrations below the limit detection.

In future studies, detection and discrimination capabilities will be proven in real industrial environments for personal air quality monitoring applications. Also, the durability of the sensors and the influence of humidity/temperature will be tested. Future revisions of the printed circuit board will include other sensors to improve detection. Other future investigation lines on this device will create pollution maps surrounding citizens who carry the device. Furthermore, studies on how contamination/dirt in the case affects the measurements will be performed as they could probably negatively affect the measurements. Last but not least, the device could be used for the detection and discrimination of odors.

## Figures and Tables

**Table 1:** Environmental Limit Values for some chemical agents according to the Spanish National Institute for Safety and Health at Work.

Chemical Agent	ELV-DE (ppm)	ELV-SE (ppm)
Toluene	50	100
Xylene	50	100
Ethylbenzene	100	200
Carbon dioxide	5000	-
Methane	1000	-

**Table 2:** AQI limits and their meaning.

AQI Value	Color	Hygienic Rating	Exposure Limit
5	Red	Situation not acceptable	hours
4	Orange	Major objections	<1 month
3	Yellow	Some objections	<12 months
2	Green	No relevant objections	No limit
1	Blue	No objections	No limit

**Table 3:** Modules used in the smartwatch and their specifications.

Sensor	Manufacturer		Dimensions (mm^3^)	Signals
BME688	Bosch	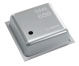	3.0 × 3.0 × 0.9	Temperature (°C), relative humidity (%), pressure (mmHg), gas resistance (Ω)
SGP40	Sensirion	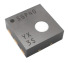	2.44 × 2.44 × 0.85	Gas resistance (Ω)
ENS160	ScioSense	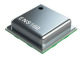	3.0 × 3.0 × 0.9	Gas resistances (Ω), TVOCs (ppm), eCO_2_ (ppm), AQI

**Table 4:** Gas concentration and temperatures.

Gas	Concentration (ppm)	Temperature (°C)
Toluene	6	61
Xylene	8	81
Ethylbenzene	10	95

**Table 5:** Variables measured by the sensors.

Sensor	Variables
ENS160	Resistance (Var3, Var4, Var5), AQI (Var6), TVOC (Var7), eCO_2_ (Var9)
SGP40	Resistance (Var8)
BME688	Temperature (Var1), Relative Humidity (Var2), Resistance (Var10)

**Table 6:** Linear regression values obtained on CO_2_ measurements and LOD.

Sensor	Equation	R^2^	LOD (ppm)
R_4_ ENS160	R = 0.0051103C + 0.1279	0.941	576.96
SGP40	R = 0.00031215C − 0.0098129	0.991	94.38
BME688	R = 0.0076506C − 0.74995	0.979	93.59
R_1_ ENS160	R = 0.00011446C − 0.01644	0.976	358.2911
R3 ENS160	R = 0.0001677C − 0.0096597	0.985	571.043

**Table 7:** Linear regression values obtained on CH4 measurements and LOD.

Sensor	Equation	R^2^	LOD (ppm)
R_4_ ENS160	R = 0.035108 − 0.035317	0.982	101.74
SGP40	R = 0.0006708C + 0.00016605	0.974	52.0404
BME688	R = 0.024595C + 1.1585	0.94	44.67
R_1_ ENS160	R = 0.00019115C − 0.0098562	0.961	295.80
R_3_ ENS160	R = 0.00043565C − 0.025474	0.959	222.55

## Data Availability

Data of the sensors will be available upon request.
